# Recent Advances in the Development of Smart and Active Biodegradable Packaging Materials

**DOI:** 10.3390/nano11051331

**Published:** 2021-05-18

**Authors:** Mahmood Alizadeh Sani, Maryam Azizi-Lalabadi, Milad Tavassoli, Keyhan Mohammadi, David Julian McClements

**Affiliations:** 1 Food Safety and Hygiene Division, School of Public Health, Tehran University of Medical Sciences, Tehran 1417614411, Iran; saniam7670@gmail.com; 2 Research Center for Environmental Determinants of Health (RCEDH), Kermanshah University of Medical Sciences, Kermanshah 6719851552, Iran; maryamazizi766@gmail.com; 3 Department of Food Science and Technology, Faculty of Nutrition and Food Sciences, Tabriz University of Medical Sciences, Tabriz 5166614711, Iran; mtavassoli2006@gmail.com; 4 Department of Clinical Pharmacy, Faculty of Pharmacy, Tehran University of Medical Sciences, Tehran 1417614411, Iran; Keyhanmohammadi72@yahoo.com; 5 Department of Food Science, University of Massachusetts Amherst, Amherst, MA 01003, USA

**Keywords:** smart materials, active packaging, colorimetric indicators, biodegradability, biocomposite films

## Abstract

Interest in the development of smart and active biodegradable packaging materials is increasing as food manufacturers try to improve the sustainability and environmental impact of their products, while still maintaining their quality and safety. Active packaging materials contain components that enhance their functionality, such as antimicrobials, antioxidants, light blockers, or oxygen barriers. Smart packaging materials contain sensing components that provide an indication of changes in food attributes, such as alterations in their quality, maturity, or safety. For instance, a smart sensor may give a measurable color change in response to a deterioration in food quality. This article reviews recent advances in the development of active and smart biodegradable packaging materials in the food industry. Moreover, studies on the application of these packaging materials to monitor the freshness and safety of food products are reviewed, including dairy, meat, fish, fruit and vegetable products. Finally, the potential challenges associated with the application of these eco-friendly packaging materials in the food industry are discussed, as well as potential future directions.

## 1. Introduction

Foods are packaged for a number of reasons, including to protect them from their environments, improve their quality and safety, increase their shelf life, and facilitate their handling, storage, and transport [1,2]. Traditionally, the packaging materials used for this purpose have been fabricated from synthetic polymers, such as polyamide, polypropylene, polyethylene terephthalate, ethylene vinyl alcohol, polystyrene, and polyvinylchloride. These synthetic polymers are particularly suitable for producing packaging materials because of their beneficial physicochemical and functional attributes, such as mechanical robustness, pliability, optical traits, and barrier properties [1]. As a result, their industrial production has continued to rise over the past few decades, with around 320 million tons currently being produced each year [1,3]. However, the widespread use of synthetic plastics for this purpose has undesirable environmental consequences, since this type of packaging material can persist in the environment for extended periods and can form microplastics or nanoplastics when it degrades that contaminate water, soil and food [1,3].

**Figure 1:**
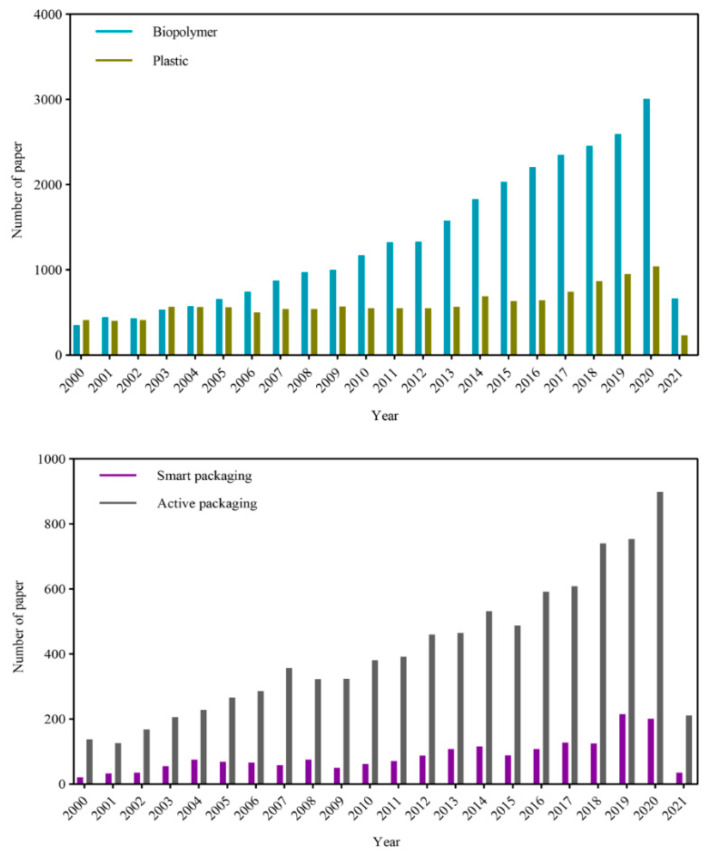
Trends in the number of scientific articles published on biopolymer-based versus synthetic plastic-based packaging materials (upper graph) and on smart packaging versus active packaging materials (lower graph). The search was carried out using Scopus and Web of Science in March 2021.

For these reasons, there has been growing interest in using natural polymers, such as polysaccharides and proteins, often in combination with other natural components (such as lipids, phospholipids, surfactants, or natural nanoparticles), to fabricate biodegradable packaging materials [4,5,6]. Indeed, the increasing research activity in this area can be seen from the number of scientific articles published on packaging materials made from biopolymers versus those made from plastics (Figure 1). The utilization of biopolymers for this purpose is often advantageous because they are more biodegradable, sustainable, and environmentally friendly than synthetic polymers [3,7,8]. In particular, biopolymer-based films can easily be degraded by microorganisms and some inorganic compounds in the environment [9,10,11]. A wide variety of biopolymers have been explored for this purpose, either alone or in combination, including cellulose, chitin, chitosan, pectin, agar, alginate, carrageenan, gelatin, zein, and whey protein [8,12,13]. One of the major challenges associated with the development of biodegradable packaging materials from biopolymers is to create films that have mechanical, optical, and barrier properties that match those normally provided by synthetic polymers [14,15]. For instance, biodegradable films may breakdown when they come into contact with moist foods or environments for extended periods, thereby losing their desirable functional attributes [9,10,11]. Researchers are therefore examining new biopolymers and their combinations in an attempt to overcome these problems. Biopolymer-based packaging materials with good functional attributes can often be prepared in the laboratory, but it is usually difficult to achieve this economically on a large-scale, which currently limits their commercial application. 

Many researchers are attempting to extend the functional performance of biopolymer-based packaging materials by creating active and/or smart films. Active packaging materials contain additives, such as antioxidants or antimicrobials, that can improve the quality, shelf-life or safety of foods by inhibiting chemical reactions or microbial growth [15]. Smart packaging materials are designed to respond to a specific trigger, such as a change in pH, temperature, moisture content, gas levels, light exposure, chemical composition, or enzyme activity [16,17]. For instance, they may contain a natural pigment that undergoes a color change in response to one of these triggers, which can then be used to report alterations in the ripeness, quality, or safety of a food [11]. Alternatively, the packaging material may respond to a trigger by releasing active ingredients, such as antioxidants or antimicrobials, that then diffuse into the food and protect it. 

**Figure 2:**
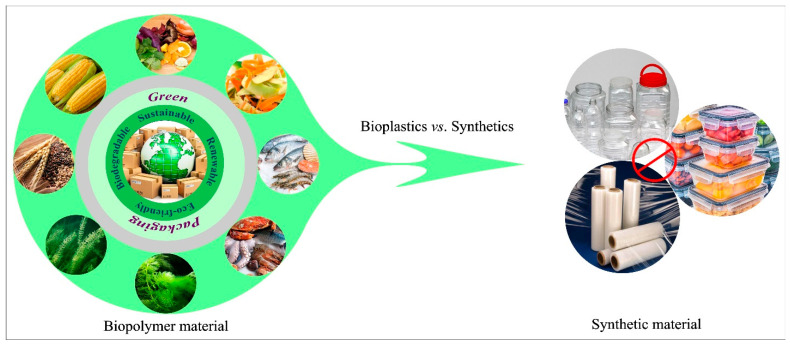
Comparison of the properties of biopolymer-based and synthetic plastic-based packaging materials.

An important advantage of using biopolymers to create packaging materials is that waste streams from the food industry can be converted into value-added functional ingredients, thereby reducing waste, increasing sustainability, and improving economic viability [18]. Many of the by-products generated by the food industry are currently used either as animal feed or simply discarded, leading to waste and pollution [19,20]. Examples of these by-products include tomato pulp, vegetable peels, fruit peels, pruning waste, and slaughterhouse waste [10]. Many of these by-products are rich sources of polysaccharides, proteins, and/or lipids, as well as other functional ingredients such as antimicrobials, antioxidants, and pigments, and are therefore a suitable source of value added ingredients [9,11]. Some of the potential advantages of biopolymer-based packaging materials over plastic ones are highlighted in Figure 2.

In this article, we review recent developments in the design and formulation of smart and active biopolymer-based food packaging materials, including discussions of the proteins, polysaccharides, and lipids that can be used for this purpose, the fabrication methods available, as well as the potential application of these materials in the food industry.

## 2. Overview of Biodegradable Packaging Materials

**Figure 3:**
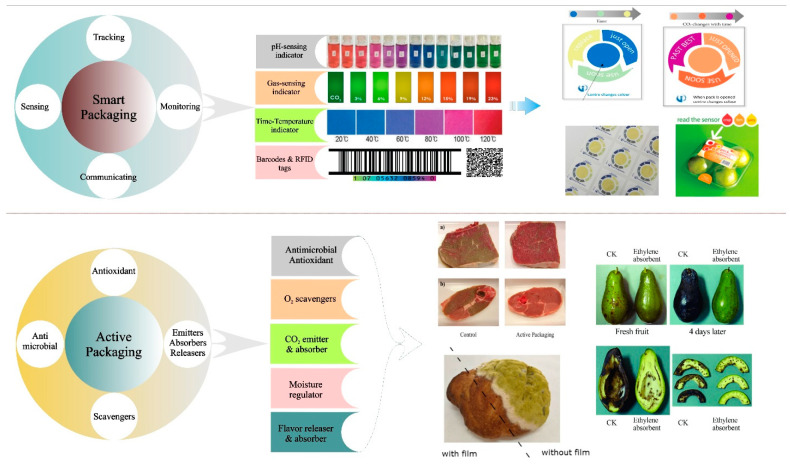
Characteristics, classification, and application of smart and active packaging materials.

Food packaging is used to protect food products from physical, chemical, or biological stresses in their environment, thereby improving their quality and extending their shelf life. A variety of packaging materials have traditionally been used for this purpose, including plastic, glass, metal, paper, wood, and textiles [1,2]. As mentioned earlier, some of the most widely used of these packaging materials, particularly plastics, cause considerable environmental damage during their manufacture and after their disposal. For this reason, there has been great interest in developing biodegradable forms of packaging materials that are more sustainable to produce, that rapidly decompose after disposal, and that do not cause as much environmental pollution [21]. These packaging materials can be constructed from biodegradable film-forming materials such as proteins, polysaccharides, and lipids. Moreover, their functional performance can be enhanced by incorporating organic or inorganic nanoparticles or nanofibers [22,23]. For instance, nano-forms of clay, iron oxide (Fe_2_O_3_), titanium dioxide (TiO_2_), silver (Ag) and zinc oxide (ZnO) can be used (inorganic nanoparticles), as well as nano-forms of chitin and cellulose and their derivatives (organic nanoparticles) [24,25,26,27,28]. The resulting nanocomposites often have enhanced technofunctional characteristics such as improved optical, mechanical and barrier properties, as well as some novel functional attributes, such as antimicrobial and antioxidant activities, that can prolong the shelf life of packaged foods [29,30,31]. Moreover, it is possible to incorporate sensing materials into biodegradable films to provide information about the quality, freshness, or safety of packaged foods. Table 1 and Figure 3 show the main characteristics of active and smart biodegradable packaging materials. 

In the remainder of this section we highlight a number of the most commonly used biodegradable materials that can be used to assemble packaging materials suitable for use in the food and other industries.

### 2.1. Biodegradable Materials

Biodegradable materials for constructing packaging materials can be obtained from plant, animal, or microbial sources. It is important that these materials can be produced economically and sustainably, and that they quickly degrade when disposed of in the environment, usually as the result of natural chemical or biochemical processes [51]. In this section, we provide a few examples of edible materials that can be used to fabricate biodegradable packaging materials.

#### 2.1.1. Proteins

##### Dairy Proteins

Dairy proteins, such as casein and whey protein, have been shown to be capable of forming biodegradable packaging materials. Caseins, which come in various types (including α_S1_, α_S2_, β, and κ caseins), make up around 80% of the proteins in milk [25,41]. These proteins are fairly flexible proteins that tend to aggregate around their isoelectric point (pH 4.6), which is important for many of their functional attributes. In the food industry, these proteins are usually available in the form of powdered calcium or sodium caseinate ingredients, which are formed by adding Ca(OH)_2_ or NaOH to casein solutions, respectively [52]. Edible films have been formed from caseinate that have favorable mechanical and optical characteristics [53]. Whey proteins, which also come in various types (including β-lactoglobulin, α-lactalbumin, bovine serum albumin, and immunoglobins), make up around 20% of the proteins in milk [52]. They are globular proteins that have also been shown to be effective at forming films due to their good gelling properties. For instance, films made from whey protein isolate (WPI) have been reported to have good mechanical and oxygen barrier properties under low and intermediate relative humidity (RH) conditions [54]. However, these films exhibited poor water vapor barrier properties, which limits their application as packaging materials for many foods. The formation of films with appropriate functional attributes requires careful control of the denaturation, association, and crosslinking of the whey proteins [55,56]. Typically, films made from milk proteins tend to be relatively soft, smooth, tasteless, and clear, which is desirable for many applications. Moreover, they can also be made to have antimicrobial and antioxidant activity by encapsulating functional additives within them [57]. One of the main challenges of this kind of packaging material is their poor resistance to moisture transport and their fragility.

##### Meat Proteins

Gelatin is one of the most commonly used meat proteins for forming biodegradable films. It is isolated from waste products of the meat industry, such as the collagen-rich bones, skin, tendons, and hooves of animals [58]. Typically, collagen is converted to gelatin by heating in a strong acid or alkaline solution at high temperatures (e.g., 80 °C) [59]. The gelatin obtained from this process is purified and then converted into a powdered form that is used as a functional ingredient in the food and other industries. Gelatin exists as a random coil molecule at high temperatures but undergoes a coil-to-helix transition when it is cooled below a critical transition temperature. The helices formed may then act as crosslinking points between different gelatin molecules due to hydrogen bonding. At sufficiently high concentrations, the gelatin molecules form a 3D network that leads to solid-like properties. Gelatin gels are typically formed by heating a gelatin solution above the coil-to-helix transition temperature (typically around 20–30 °C for terrestrial animals and lower for fish), and then cooling and drying the solution, which increases the protein concentration and promotes crosslink formation [60,61]. Gelatin films can be formed with thicknesses and mechanical properties suitable for use as food packaging materials, but they often have poor barrier properties, especially against water vapor transport [62,63], which limits their practical applications.

##### Plant Proteins

Many different kinds of plant protein are available to produce biodegradable films, including those isolated from zein, gluten, soybeans, nuts, peas, and sunflower [64]. Zein is a hydrophobic corn protein that is insoluble in water but soluble in concentrated alcohol solutions, which is important for the formation of edible films [65]. Previously, zein has been used as a constituent of packaging materials for various foods [66,67]. The proteins isolated from soybeans have also been shown to be suitable for forming edible films [68], which is often carried out using film casting or baking methods [69]. Smooth and stretchable edible films can be formed from soy proteins that have good mechanical properties, but again their water barrier properties tend to be poor [70]. The water barrier properties of soy films can be improved by incorporating hydrophobic additives into them, such as stearic acid, but this also modulates their optical and mechanical properties [71]. Other additives, such as glycerol, gellan gum or κ-carrageenan, have also been shown to improve the functional performance of soy films [72]. 

#### 2.1.2. Polysaccharides

Polysaccharides such as starch, cellulose, chitin, chitosan, and hydrocolloid gums, have also been used as components to construct biodegradable films [4,6]. These polysaccharides differ in their molecular characteristics, which alters the physicochemical and functional attributes of the packaging materials constructed from them

##### Starch

Starch is widely used because of its relative cheapness, abundance, biodegradability, and renewability [73]. In nature, starch molecules are packed into small granules (around 1 to 20 μm) that consist of amylose and amylopectin molecules organized into concentric amorphous and crystalline rings [74]. Edible films made entirely from starch have a high water vapor permeability and weak mechanical properties, which limits their usage [75]. For this reason, researchers have examined the impact of incorporating other additives to improve their functional performance. For instance, starch has been combined with polyvinyl alcohol to produce a film with good barrier properties against water, thereby extending its potential for commercial applications as a food packaging material [76]. 

##### Cellulose

Cellulose is the most abundant source of functional polysaccharides in nature, which is usually obtained from wood or cotton using acid hydrolysis processes [77]. Cellulose and its derivatives, such as methylcellulose (MC), hydroxypropyl methylcellulose (HPMC), and carboxymethyl cellulose (CMC), have been widely explored for their potential in forming biodegradable films [78,79]. For instance, films with good mechanical and water solubility characteristics have been produced using CMC [80]. However, other studies have reported that cellulose-based films act as poor water vapor barriers, which limits their application in foods [81].

##### Chitin and Chitosan

Chitin is the second most abundant polysaccharide found in nature, while chitosan is produced from chitin using controlled de-acetylation reactions [51]. Chitin and chitosan have both been shown to be capable of forming biodegradable films that can be used to increase the shelf life of food products [82]. Typically, the films formed by chitin are mechanically weaker and have worse barrier properties than those formed by chitosan. As with other biopolymers, the functional performance of chitin and chitosan films can be improved by combining them with proteins or other polysaccharides, or by incorporating other functional additives [28,42]. The fact that both chitin and chitosan naturally exhibit antimicrobial activity is useful for the development of active biodegradable films that can increase the shelf life of foods [42,51].

##### Hydrocolloid Gums

A variety of edible hydrocolloid gums can be used to form biodegradable packaging materials. Pectin is an anionic polysaccharide consisting of a linear anionic chain with neutral side chains attached to certain regions [10,13]. Commercial pectin ingredients are typically isolated from apple, citrus fruit, or sugar beet. Pectin is widely used in the food industry as a stabilizer, thickening agent, gelling agent, and film former [83]. Studies have shown that pectin can form films that are relatively strong and have good resistance to oxygen diffusion, but are fragile and have poor resistance to water diffusion [84]. Pectin films have been shown to be able to protect foods with relatively low water activities [85]. They have also been reported to increase the shelf life of a wide range of fruits and vegetables, including apple, apricot, avocado, berries, guava, chestnuts, melon, peach, walnuts, papaya, tomato, and carrot [86]. Pectin is often preferred for these applications because it can be naturally derived from fruits and vegetables. Nevertheless, numerous other kinds of hydrocolloid gums can also be utilized to create biodegradable films because of their ability to form crosslinks with each other, including agar, alginate, carrageenan and gum arabic [87,88]. 

#### 2.1.3. Lipids 

A number of lipids can be used to assemble biodegradable films, either in isolation or in combination with other components, including monoacylglycerols, diacylglycerols, triacylglycerols, phospholipids, free fatty acids, and waxes [89,90,91]. Lipid-based films have advantages for creating a glossy surface appearance, retaining moisture in foods, and reducing water permeability [92,93]. For instance, films produced from palm fruit oil have been reported to be transparent and have good water barrier properties [94]. Sunflower oil-based films have been used to coat hamburgers, which were shown to improve their quality by controlling oxygen and water vapor permeability [95]. Essential oils (EOs) isolated from the peels of citrus fruit (such as lemon, mandarin, and orange) have been incorporated as functional ingredients into methylcellulose and chitosan films to enhance their functionality [96]. Antimicrobial essential oils from cinnamon, allspice, and clove bud have also been incorporated into edible films to protect apples during storage [97]. In many cases, lipids are converted into an oil-in-water emulsion by homogenizing them with an aqueous solution containing an emulsifier prior to incorporating them into biopolymer-based films. The composition, size, concentration, and interfacial properties of the lipid droplets used impacts the mechanical, optical, barrier and other functional attributes of the films formed, and should therefore be optimized for each application [94].

## 3. Fabrication of Packaging Materials

In this section, a brief overview of the various methods commonly used to produce biodegradable packaging materials is given, including casting, electrospinning, extrusion, and compression methods [51,98,99]. The casting method is the most widely used in research laboratories for the small-scale production of biodegradable packaging materials from food-grade ingredients [51]. Typically, the film-forming biopolymers are dissolved within a solution and then any functional additives are incorporated (such as plasticizers, nanoparticles, nanofibers, phytochemicals, or emulsified lipids). These mixtures are then cast in petri dishes and placed in a vacuum oven to remove the water or other solvents (e.g., 50 °C for 48 h). The resulting films are then often stored in desiccators at a fixed relative humidity before they are characterized and utilized [100]. Although this method is widely used in scientific research, it is typically unsuitable for the large-scale production of packaging materials. However, it is useful for identifying potential formulations that could be produced using other methods once a suitable scale-up procedure has been established. 

Electrospinning processes are also commonly used in research laboratories but may also be used on an industrial scale [101]. In this case, a solution containing the film-forming biopolymers and additives is placed into a syringe. A high voltage is applied between the syringe tip and a collection plate. The mixture is pulled out from the syringe tip and forms a thin stream, which is dried as it passes through the intervening air. As a result, fibers are deposited onto the collection plate, which can then be further dried by holding them at an elevated temperature [102]. This method tends to produce highly porous fiber films containing interconnected pores with a high specific area [103,104]. The composition and structure of these films can be controlled by changing the ingredients and operating conditions used [104]. The delicate fibrous mats produced by electrospinning may be suitable for some packaging applications but are less suitable for forming thin films [99,104]. 

Extrusion methods can be used to make biodegradable packaging materials on a small or large scale. They involve applying high temperatures, pressures, and shear forces to mixtures of biopolymers and other additives to blend and plasticize them. The resulting material is then forced through a narrow die with the required shape. The nature of the films produced depends on many factors, including the biopolymers, additives, and processing conditions used, including the operating temperatures, pressures, and shearing rates [105]. Glycerol is often used as a food-grade plasticizer because of its good thermal stability. Extrusion is particularly suitable for the large scale production of packaging materials because it can be carried out as a continuous process at large scales [106].

Biodegradable films can also be produced at small and large scales using compression molding methods [51,98]. In this process, the film-forming biopolymers and other additives are mixed together and then placed into a suitable mold. The film may then be formed by compression of these mixtures to promote curing, that is, crosslinking of the biopolymers. In cold compression, the curing procedure takes place at room temperature, while in hot compression it takes place by applying heat to the mold during compression [107]. 

The selection of an appropriate fabrication method depends on the nature of the ingredients used, the desired attributes of the final packaging materials, and the amount of material that needs to be produced. The casting method can be used with most biopolymers albeit at a small scale, but the other three methods can only be used for certain types of biopolymers. For example, the electrospinning method can only be used for electrically charged biopolymers that can be pulled through a nozzle. The extrusion method is unsuitable for biopolymers that chemically degrade at high temperatures, pressures, or shear rates. The compression methods are only suitable for biopolymers that set when they are compressed or compressed/heated [51,98,99]. 

## 4. Active Packaging Materials

Active food packaging materials are designed to have functional attributes that go beyond the normal optical, mechanical, and barrier properties of conventional packaging materials. For instance, they may be designed to inhibit microbial growth, to retard undesirable chemical reactions, or to control enzyme activity, thereby extending the shelf life of foods [108,109]. Typically, this is achieved by incorporating antimicrobials and/or antioxidants agents into the packaging materials, with a particular emphasis on the utilization of natural botanically-derived functional ingredients for clean labeling purposes [7,25,110,111,112]. One of the potential advantages of active packaging materials is that the antioxidants and antimicrobials are located within the film, rather than within the food, which may reduce the amount of these additives required to protect the food during storage, as well as reducing the amount ingested [108,109,113,114].

The additives incorporated into active packaging materials may increase the shelf life of packaged foods by a range of different mechanisms depending on their characteristics. Some of the most common additives that exhibit antimicrobial or antioxidant properties that are used for this purpose include macronutrients (such as specific protein or carbohydrate molecules), inorganic nanoparticles (such as Ag, TiO_2_, ZnO, and clay), organic nanoparticles (such as lipid-, protein-, or carbohydrate-based nanoparticles), essential oils or other extracts from plants (such as thyme oil or tea extract), and phytochemicals (such as curcumin, quercetin, or anthocyanins). A number of these additives are discussed in more detail in the following sections.

### 4.1. Antioxidants

The reason for incorporating antioxidants into food packaging materials is to inhibit oxidation reactions in foods, particularly degradative reactions that involve lipids or proteins [111,115]. Typically, antioxidants inhibit oxidation by neutralizing singlet oxygen, reducing hydrogen peroxide, quenching free radicals, or chelating transition metal ions [108,115,116,117]. Botanical extracts from a wide range of plants including saffron, garlic, cabbage, potatoes, tomatoes, and strawberries have been used as natural antioxidant additives in active packaging materials [108,118,119,120]. These extracts contain various classes of molecules that can exhibit antioxidant activity [113,121]. For instance, epigallocatechin and epigallocatechin gallate were shown to have the highest antioxidant activity in green tea extracts [122]. Another study examined the antioxidant activity of various kinds of plant extracts in gelatin films, including ginger, grape seed, green tea, and ginkgo leaf extracts using a DPPH radical scavenging assay [123]. This study showed that ginkgo leaf extracts had the most potent antioxidant activity.

Anthocyanins are water-soluble pigments that are naturally abundant in many plants and their by-products, including flowers, cereals, vegetables, and fruits [108]. These natural phenolic compounds also have excellent antioxidant and antimicrobial properties [14,16]. Anthocyanins have been shown to play promising antioxidant roles in active packaging materials as a reducing agent and an oxygen suppressor [14,108]. The potency of these antioxidants depends on various factors, including the type of anthocyanin used, the composition of the biopolymer matrix, and the method of film preparation [14,115].

Essential oils are another group of botanical compounds that have strong antioxidant and antimicrobial properties, which consist of a complex mixture of phenolic, terpene, and terpenoid compounds [113,124]. Essential oils have been classified as Generally Recognized as Safe (GRAS) and so can be used in active food packaging materials as functional ingredients [125,126]. Essential oils such as carvacrol, cinnamon, thyme, rosemary, citrus, and tea oils can be extracted from botanical sources by distillation [109,127]. Incorporation of these essential oils into packaging materials can enhance their physicochemical and mechanical properties, as well as their antimicrobial and antioxidant properties [26,125]. Carvacrol is the main antimicrobial component found in oregano oil, which has been shown to increase the permeability of microbial cell walls, thereby resulting in their death [108]. A synergistic antimicrobial effect against strawberry gray mold has been reported when carvacrol was used in combination with thymol in clay/polymer nanocomposite films [128]. Essential oils have also been used in combination with other kinds of inorganic nanoparticle additives in biopolymer packaging materials, such as those comprised of titanium oxide (TiO_2_), zinc oxide (ZnO), and silver (Ag), to improve their antimicrobial and antioxidant properties [109,113,125]. A combination of rosemary oil and TiO_2_ nanoparticles incorporated into a biopolymer packaging material has been reported to significantly reduce lipid oxidation and microbial growth [113]. Incorporating inorganic nanoparticles into packaging materials has been shown to eliminate the characteristic odors associated with essential oils. For instance, it was reported that the incorporation of carvacrol oil into packaging materials led to an unacceptable odor [129], which could be reduced by also incorporating ZnO NPs [130,131]. 

### 4.2. Antimicrobials

The incorporation of antimicrobial substances into active packaging materials is advantageous because it can be used to inhibit the growth of spoilage or pathogenic microbes [12,108]. Natural antimicrobial substances, such as essential oils (cloves, oregano, thyme, rosemary oils) and plant extracts (barberry, saffron, potatoes, strawberries, garlic, tomatoes, lettuce, and cabbage extracts) can be included into biodegradable packaging materials [42,79,108,119,132,133]. These antimicrobial substances may remain in the packaging materials or they may diffuse into the foods during storage [108,114]. Ideally, the rate at which the antimicrobials move into the foods can be controlled so as to prolong their activity. Antimicrobial substances can be immobilized on the surfaces of package materials or they can be incorporated throughout them [108,109]. Some natural antimicrobial substances are sensitive to heat, so their antimicrobial activity may be lost during thermal processing. In this case, non-thermal fabrication methods such as electrospinning, solvent evaporation, or casting methods should be used to prepare this type of packaging material [131]. Anthocyanins have been shown to have good antimicrobial properties in active packaging materials, which has been attributed to their ability to penetrate the cell membranes, inhibit extracellular enzymes, and breakdown the cytoplasmic membranes of microorganisms [110].

Various other kinds of natural antimicrobial agents have been investigated for their potential incorporation into active packaging materials [134]. For instance, incorporating eugenol into gelatin films increased their antibacterial activity, with a reduction of microbial growth, as expressed as colony forming units (CFU), of around 2.5 log units for *S. aureus* and 3 log units for *E. coli* compared to the control group [135]. In another study, it was shown that incorporation of tomato extract and itaconic acid into films comprised of chitosan and poly(vinyl alcohol) reduced improved their antibacterial activity against *P. aeruginosa* and *S. aureus* [136]. Active packaging materials have also been developed to reduce contamination by other kinds of microbes, including viruses and fungi [137]. For example, the incorporation of a tea extract into chitosan-based films was shown to increase their antiviral activity against murine norovirus (MNV-1) [138]. In another study, incorporation of silver into polylactic acid films was shown to increase their activity against feline virus (FCV), another surrogate for human norovirus, with no infectious FCV being detected in lettuce samples incubated at 4 °C for 6 days [139]. Packaging materials with antifungal activity have also been developed. For instance, incorporating cinnamaldehyde into gliadin-based films reduced food spoilage by inhibiting the growth of *Aspergillus niger* and *Penicillium expansum* on bread and shredded cheese [140]. 

### 4.3. Gas Controllers

Oxygen molecules can permeate through food packaging materials and accelerate oxidation and discoloration reactions in packaged foods [141]. Consequently, it is important to have methods to control oxygen levels in foods. Oxygen-scavenging agents, such as iron acids, sulfites, catechols, ascorbic acid, unsaturated hydrocarbons, palladium, tocopherols, and enzymes (glucose oxidase), can be used as oxygen depleting agents [141]. Other methods of inhibiting the adverse effects of oxygen include the use of botanical substances such as flavonoids, phenolics, salicylic acid, and gallic acid [124,142]. These compounds can sometimes be obtained from waste streams of food processing operations, which improves the economics and sustainability of the food supply [143]. To inhibit oxygen, a substance should have a number of desirable structural features, including the presence of carbon-carbon and carbon-oxygen double bonds, and the presence of hydroxyl groups [108,141,143]. 

Ethylene gas (C_2_H_4_) is naturally produced by fruits and vegetables during respiration [144], which impacts their ripening, color, texture, and quality [108]. It is therefore important to be able to control the ethylene gas content in packaged produce during storage. In general, ethylene levels can be controlled by incorporating substances that absorb, oxidize, or decompose the gasses produced by fruits and vegetables [145]. Compounds that can remove and adsorb ethylene gas include potassium permanganate (KMnO_4_), clay, palladium, activated carbon, and titanium dioxide [144]. These kinds of additives can be used individually or in combination to obtain synergistic effects. For instance, nanocomposite films have been prepared from chitosan, TiO_2_ nanoparticles, and black plum peel extract, which were shown to have good antioxidant, antimicrobial, and ethylene scavenging properties [146].

In general, the optimization of an active packaging material for a particular application depends on several factors, including the water activity, composition, and pH of the food product, as well as the temperature and relative humidity of the environment during storage [147]. In addition, the packaging materials must be formulated so that they have desirable physicochemical and functional properties, such as optical, mechanical, barrier, sensory, and other attributes. The results of some recent studies on the development of active packaging materials are summarized in Table 2, while the growing number of articles published in this area is highlighted in Figure 1.

## 5. Smart Packaging Materials

Smart packaging materials are designed to respond in a particular manner when there is some change in the system (such as a change in quality, safety, or maturity of a packaged food), or to provide an indication of these changes [16,160]. As a result, these smart packaging materials can play an important role in improving food quality and safety management [16,161]. Examples of the several smart packaging materials that have been reported in the literature are highlighted in Table 3. In this section, some of the most common sensors that have been developed for application in smart packaging materials suitable for food applications are described.

### 5.1. pH Indicators

This kind of indicator provides a measurable change when there is a significant alteration in the pH of a packaged food. These pH changes may be caused by enzymatic activity, chemical reactions, or microbial growth in foods, and so pH sensors can provide an indication of alterations in food quality or safety [16,28]. Due to increasing demand from consumers for clean-label products, the use of natural pigments is usually preferred over synthetic dyes [178,179]. Several kinds of natural pigments undergo specific color changes in response to an alteration in the pH of the surrounding medium. This type of colorimetric pH-sensor, which is also referred to as a halochromic sensor, is typically based on an exchange of protons (H^+^) between the pigments and their environment [180]. Some colorimetric indicators can also give a color change in response to the presence of specific volatile compounds, which can also provide an indication of alterations in food quality [17,181,182,183].

**Figure 4:**
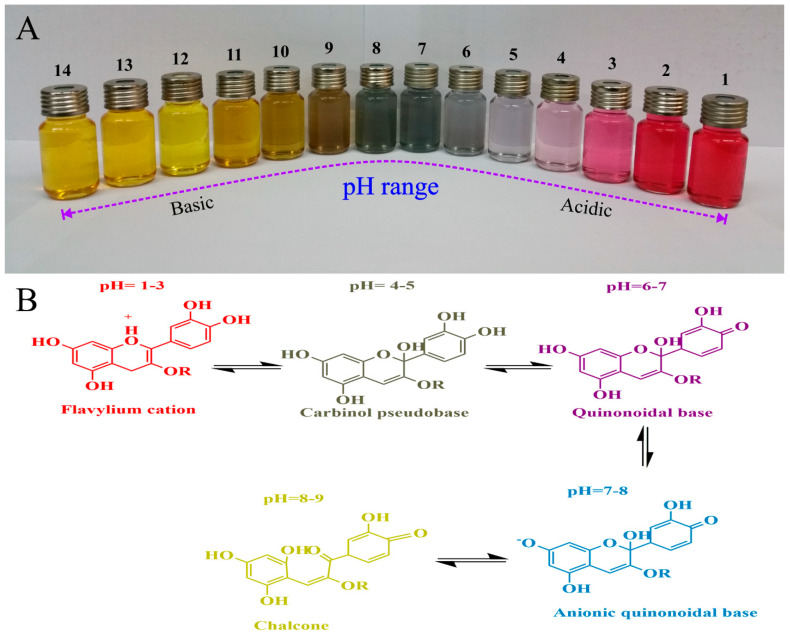
Solution color variations (**A**), and structural transformation of saffron petal anthocyanins in various buffer solutions (**B**), Reprinted from [42], copyright 2021, with permission from Elsevier.

pH-sensitive indicators have been developed using anthocyanins derived from various botanical sources, including saffron petal [42], black rice bran [184], hibiscus [185], purple corn [186], black soybean seed coat [44], purple onion peel [187], roselle [163,188], red barberry [28,79], blueberry [20,43], purple sweet potato [189,190], red cabbage [132,191], as well as from carotenoids [192,193], betalains [194,195] and chlorophylls [196]. The response of these natural pigments to pH changes depends on their molecular structure, as well as on environmental conditions, such as temperature, oxygen levels, and light exposure [197]. Anthocyanins are currently the most widely used natural pigments in smart packaging applications because they exhibit characteristic color changes over a broad range of pH values [16], changing from red (strongly acidic) to purple (mildly acidic) to violet (neutral) to blue (mildly alkaline) to green (moderately alkaline) to yellow (strongly alkaline) with an increase in pH (Figure 4). Under acidic conditions, the predominantly red color is caused by the flavylium cation (oxonium form). As the pH is increased, a number of different anthocyanin species with different absorption spectra are present in equilibrium with each other. In mildly acidic and neutral conditions, the carbinol pseudo-base and quinoidal base (hemiketal form) dominate, respectively. Under mildly alkaline conditions, the anionic quinoidal base species appears. Under strongly alkaline conditions, anthocyanins are chemically unstable and degrade into a chalcone species that has a green/yellow color [197,198]. Consequently, anthocyanins can be used as sensors over a wide pH range because they have different characteristic colors under acidic, neutral, and alkaline conditions. These anthocyanins can therefore be incorporated into biopolymer-based smart packaging materials as pH sensors to monitor changes in the quality or spoilage of foods. This kind of smart packaging material has been shown to be useful for detecting quality changes in a number of food applications including, pork [162,164,199], shrimp [184], chicken [166,169], milk [200,201], pork, shrimp, fish [202,203,204], and Atlantic mackerel [205]. 

Other natural pigments are also available that undergo characteristic color changes when the pH is altered and so can also be used as sensors of food quality [16]. For example, carotenoids (lycopene/bixin/β-carotene) have been incorporated into polylactic acid films to monitor and control the oxidation of sunflower oil [193]. The carotenoids act as natural antioxidants that slow down oxidation but they also undergo color changes when they are oxidized, thereby providing an indication of oil quality. Betacyanin derived from dragon fruit peel has been incorporated into glucomannan/polyvinyl alcohol films as an indicator of the freshness of packaged fish [172]. The pigments changed color from purple under acidic conditions to yellow under alkaline conditions, which provided an indication of changes in fish quality. Chlorophyll has been incorporated into wheat gluten/polypyrrole films as a color indicator of pH changes related to quality [196]. 

### 5.2. Gas Indicators

Fresh fruits and vegetables produce gasses (such as ethylene) as a result of natural respiration processes, which provides a measure of their freshness and quality. Moreover, gasses (such as oxygen) may move into or out of food packages and alter the susceptibility of the foods to chemical degradation (such as oxidation). Finally, certain kinds of gasses are generated due to the action of microbes that contaminate foods, thereby providing an indication of their quality and safety. For this reason, it is important to have smart biodegradable films that can sense and indicate the presence of specific gasses [113,206]. 

Smart packaging materials have been developed that contain sensors that are sensitive to different kinds of gases (e.g., CO_2_, O_2_, H_2_S, and ethylene) and other volatile compounds (e.g., amines, ketones, and aldehydes) that provide an indication of food quality [177,200,204,207,208]. These gas sensors can be developed based on the tendency for some natural pigments to chemically degrade when exposed to certain kinds of gasses. For instance, anthocyanins degrade in the presence of ammonia (NH_3_) vapor, with the color changing from purple/violet to green/yellow as the gas concentration increases [132]. Similarly, betalains chemically degrade in the presence of oxygen, which leads to a measurable color change [209]. Moreover, many carotenoids exhibit color fading when exposed to oxygen due to oxidation reactions, and so they can be employed as oxygen sensors [210]. In principle, different natural pigments can be used to detect different kinds of gasses. Colorimetric gas indicators can be incorporated into packaging materials in a variety of ways, including adhesive labels, printed layers, or within the interior of the film [113]. These smart packaging materials can provide information much more cheaply and quickly than analytical instruments such as spectrophotometry, chromatography, mass spectrometry, or nuclear magnetic resonance methods [206]. Numerous studies have demonstrated the potential of smart packaging materials containing gas sensors to detect different kinds of gasses including oxygen [211], carbon dioxide [207,208,212], hydrogen sulfide [200,213], ethylene and volatile ammonia compounds [208,214]. As an example, a colorimetric gas-sensing indicator has been used to monitor changes in CO_2_ levels, which provides an indication of the freshness of green bell peppers [215]. The colorimetric films changed from green to orange when the fresh-cut bell peppers deteriorated. In another study, a smart colorimetric packaging material consisting of a starch/polyvinyl alcohol film loaded with roselle (*Hibiscus sabdariffa* L.) anthocyanins was shown to be suitable for monitoring changes in the freshness of silver carp (*Hypophthalmichthys molitrix*) stored at 4 °C [174]. The colorimetric label changed from purple (acidic) → pink → violet → bluish → green/yellow (basic) over time due to the formation of volatile basic nitrogen amines. In another study, smart colorimetric packaging materials consisting of tara gum/polyvinyl alcohol films containing curcumin were used to monitor changes in the freshness of shrimp by detecting the generation of NH_3_ [177]. The color of the smart indicator film reversibly changed from yellow to brown as the NH_3_ concentration increased. 

### 5.3. Time-Temperature Indicators

In general, time-temperature indicators (TTIs) are used to monitor and track the quality and safety of packaged foods during storage and distribution by determining whether they have been exposed to elevated temperatures for extended periods [113]. Smart packaging materials loaded with TTIs have been developed using natural food pigments as indicators [216,217,218,219]. The extent of the color change of these indicators depends on the temperature-time profile that the packaged food has been exposed to. These TTIs can therefore be used to obtain an indirect indication of whether a food product is likely to have deteriorated during storage [216,219,220]. TTIs have been widely used in the food packaging industry because they are relatively simple and inexpensive to design, as well as being easy to read by consumers [113]. TTIs are categorized into different groups depending on the underlying principles of the temperature-detection method: diffusion, polymerization, microbial growth, enzymatic reaction, thermochromic reaction, photochromic reaction, electronic, and surface plasmon resonance [221,222]. Sensors that depend on temperature can also be classified into different categories depending on their mode of operation: (i) *critical temperature indicators* (CTI), which report whether the food has been heated above or cooled below some specified temperature during its lifetime; (ii) *critical temperature/time integrators* (CTTI), which report whether the food has been heated above or cooled below some specified temperature for longer than a specified time; and (iii) *temperature-time indicators* (TTIs), which report the full temperature versus time profile of a food product throughout its history [113]. It is therefore important to select a temperature sensor that can provide the required response to a change in its thermal environment. Typically, a temperature sensor has an activation energy (*E_a_*) that must be overcome before there is a change from one state to another, such as a color change [113]. Temperature sensors typically follow an Arrhenius temperature dependency and it has been estimated that their activation energy should be in the range of about 10 to 40 kcal/mol [223]. A well designed temperature sensor can provide information about the expected shelf life of a food product provided there is prior knowledge about the impact of storage conditions on shelf life [113,222].

A number of researchers have examined the suitability of natural pigments as temperature sensors. Various types of anthocyanins have been shown to exhibit discoloration when the temperature exceeds about 30 °C, including those isolated from vegetable extracts [224], blue flowers [225], pomegranate juice [226], and fruits purees [227]. As an example, smart packaging materials have been developed by integrating a temperature-sensitive anthocyanin into a chitosan/cellulose matrix, which irreversibly changed color from violet to yellow when the temperature was increased from 40 to 70 °C [228]. A number of other time-temperature colorimetric indicators have been developed based on other sensor mechanisms, including microbial-based (green to red), polymer-based (colorless to blue), diffusion-based (yellow to pink), and enzymatic-based (green to yellow to red) TTIs [206,229].

## 6. Applications of Biodegradable Packaging Material

Biodegradable packaging materials have been developed to increase the quality, shelf life, and safety of many kinds of foods [230,231]. In the following section, several applications of these packaging materials are presented for different food products. In addition, Table 4 summarizes a number of previous studies on the application of smart and active packaging materials in real foods.

### 6.1. Meat and Seafood

**Figure 5:**
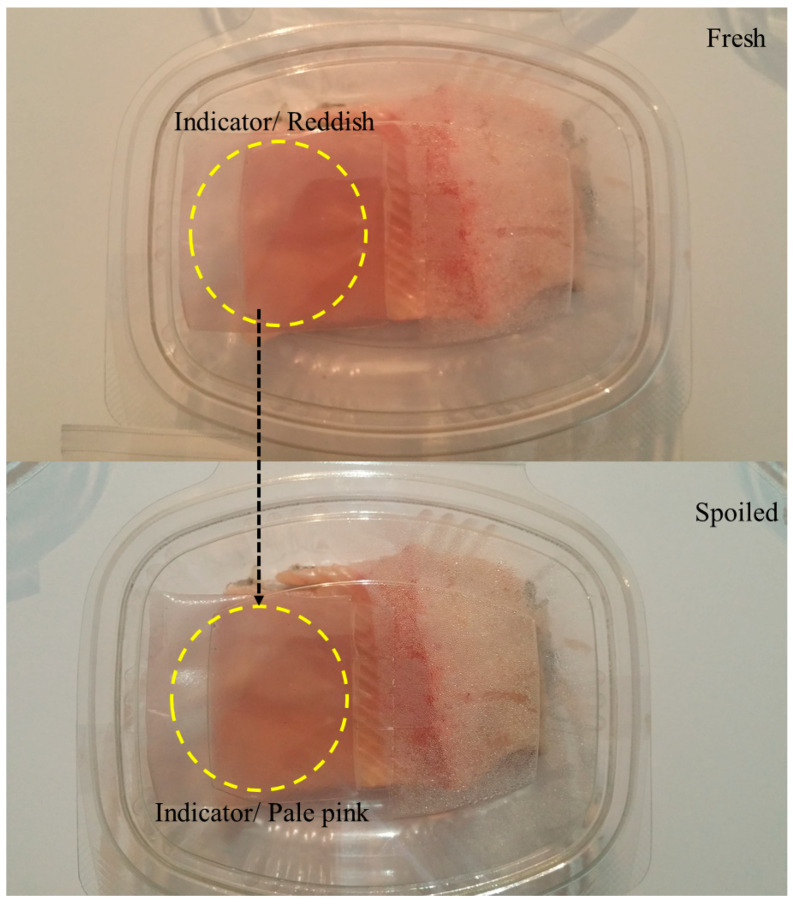
Monitoring and tracking the freshness and spoilage of fish fillet using smart halochromic film, Reprinted from [28], copyright 2021, with permission from Elsevier.

Biodegradable packaging materials have been used to extend the shelf life and improve the quality of meat products. These packaging materials are often used to control the environment around red meat so as to prevent undesirable color changes associated with myoglobin [113]. Consequently, they should have the ability to control the flow of gasses (such as oxygen) into and out of the package. In addition, active packaging materials may contain antimicrobial or antioxidant components to slow down microbial contamination or oxidation reactions, whereas smart packaging materials may contain sensors to provide insights into the quality or safety of the product (Figure 5) [245]. As an example, biodegradable packaging materials have been developed for meat applications that consist of a starch/whey protein film that includes a red cabbage extract as a natural antioxidant to inhibit lipid oxidation and improve meat quality [246]. Similarly, whey protein films have been developed that contain antimicrobial essential oils (rosemary) and titanium dioxide nanoparticles to improve the quality and shelf life of lamb meat during refrigerated storage by inhibiting microbial growth [236]. These films were reported to increase the shelf life of the meat products from around 6 to 13 days at 4 °C. In another study, smart packaging materials were prepared that consisted of κ-carrageenan films containing a botanical extract (*Lycium ruthenicum Murr*) as a color indicator, which changed color depending on the degree of spoilage of packaged shrimp [247]. A temperature-sensitive packaging material has been developed to give an indication of the quality status of fresh beef during storage [248]. Fish and other seafood products are also highly perishable foods as a result of microbial spoilage and oxidative reactions [249,250]. Active and smart packaging materials have also been shown to be effective at protecting these products, as well as at monitoring their quality during storage [28,79,112,236].

### 6.2. Dairy Products

Dairy products are nutrient-rich foods that are highly susceptible to microbial and chemical degradation during storage, thereby negatively impacting their quality attributes and safety [251]. A number of researchers have shown that active packaging materials can be used to increase the shelf life and quality of dairy products by including antimicrobial or antioxidant substances [251]. For instance, packaging materials consisting of sodium alginate films containing lemon extract were shown to inhibit the growth of spoilage microorganisms in mozzarella cheese, thereby extending its shelf life [252]. Similarly packaging materials consisting of starch films containing antimicrobial essential oils (carvacrol, linalool, and thymol) were shown to inhibit *Saccharomyces cerevisiae* growth on Cheddar cheese [253]. Smart packaging materials have also been created that consisted of starch/polyvinyl alcohol films containing anthocyanins and limonene, which changed from purple to red when the pH of pasteurized milk changed during storage [244]. In another study, smart packaging materials were developed that consisted of chitosan/PVA films containing anthocyanins from red cabbage, which provided an indication of the thermal history of milk products during storage based on color changes [118].

### 6.3. Fruits and Vegetables

Fresh and cut fruits and vegetables are highly perishable foods whose quality and safety may deteriorate during storage because of insect infestation, microbial contamination, or biochemical processes such as respiration [254,255]. Consequently, active and smart packaging materials are being developed to protect these foods during storage and transport, as well as to report on their quality status [256,257,258]. For instance, incorporating titanium dioxide nanoparticles into chitosan films was shown to increase the shelf life of tomatoes by delaying their ripening [239]. Incorporating anthocyanin-rich blackberry extracts into carboxymethylcellulose (CMC) films was also reported to increase the shelf life of cherry tomatoes [259]. Similarly, incorporating essential oils encapsulated in β-cyclodextrins into packaging materials was shown to increase the shelf life and quality of cherry tomatoes [260]. Antimicrobial packaging materials containing palmarosa essential oils or star anise were shown to increase the shelf life and reduce the growth of *Penicillium expansum* in apples [261]. 

## 7. Conclusions and Future Prospective

Smart and active packaging materials fabricated from natural materials have considerable potential in the food industry to improve the quality and safety of foods, as well as to extend their shelf-life and reduce waste. Natural pigments can be incorporated into these materials as indicators of changes in freshness, quality, or safety by undergoing color changes in response to specific alterations in pH, gas levels, or temperature. Natural antimicrobials or antioxidants can be used to extend the shelf-life of packaged foods by inhibiting microbial growth or undesirable chemical reactions. In some cases, a single additive can have multiple functions, acting as an antimicrobial, antioxidant, and sensor. The main advantage of smart packaging materials is that the freshness and safety of a product can be monitored in real-time without having to open the package. Moreover, insights into the previous history of the product can be ascertained, such as its exposure to light, oxygen, pH, or temperature changes. 

Despite their considerable potential, there are still a number of hurdles that must be overcome before the more widespread commercial use of these active and smart packaging materials in the food industry. In particular, most of the packaging materials developed so far do not meet the rigorous optical, mechanical, barrier, or stability requirements needed for commercial applications. Moreover, there is a need to develop packaging materials that can be produced economically on a large scale. In addition, there is a need to ensure that any packaging materials that are developed remain intact and perform under the wide range of environmental conditions that foods experience during their production, storage, and utilization, including changes in temperature, light exposure, relative humidity, and mechanical stresses. Clearly, further research is still required to create the next-generation of biodegradable smart and active packaging materials that are robust and commercially viable, which may involve the identification and use of new natural materials, as well as the implementation of innovative structural design and processing approaches. 

## Figures and Tables

**Table 1:** Main characteristics of active and smart biodegradable packaging films.

Polymer(s)/Biopolymer(s)	Active Material(s)	Smart/or Active Packaging	Characteristics of Packaging Films											Thermal	Ref.
			Physical				Mechanical			Barrier		Optical			
			WS	MC	WCA	Th	TS	EB	YM	WVP	OP	T600/Op	Color		
Chitosan/poly (vinyl alcohol)	Boswellic acid	Active	+	−	+	±	+	−	+	−	−	−/+	−	−	[32]
Gelatin	Grapefruit seed extract/TiO2 NPs	Active	N	N	−	+	−	+	−	+	N	−/+	+	−	[33]
Poly(lactide)/poly(butylene adipate-co-terephthalate)	Ferulic acid	Active	N	N	−	+	+	−	+	N	N	−/+	N	−	[34]
Poly(lactic acid)/poly(butylene-succinate-co-adipate) (PLA/PBSA)	Thymol EOs	Active	−	−	N	+	−	+	−	−	−	−	N	−	[35]
Starch	Yerba mate extract	Active	−	−	N	+	+	+	−	−	−	N	N	N	[36]
Poly(vinyl alcohol)/clay	Tea polyphenols	Active	−	−	±	±	+	−	N	−	−	−/+	+	N	[37]
Chitosan/gallic-acid	ZnO NPs	Active	−	−	N	+	−	+	N	−	−	−/+	N	N	[38]
Corn starch/chitosan	Grapefruit seed extract	Active	+	+	N	+	−	+	−	−	−	N	N	−	[39]
Gelatin	Silver-Kaolin NPs	Active	−	−	+	+	+	−	+	−	N	−/+	N	N	[40]
Sodium caseinate/guar gum	TiO2 NPs/cumin EOs	Active	−	N	−	+	+	±	+	±	N	−/+	N	−	[41]
Methyl cellulose/chitosan nanofibers	Saffron petal anthocyanins	Smart	−	−	N	+	+	+	−	−	N	−	+	−	[42]
Cassava starch	Blueberry residue	Smart	+	+	+	±	−	+	−	±	+	−/+	+	−	[43]
Chitosan	Black soybean seed coat extract	Smart	+	−	N	+	+	+	N	−	N	+	+	−	[44]
Gelatin	Red cabbage (*Brassica oleracea* L.) extracts	Smart	+	−	N	+	+	+	−	+	N	+	−	N	[45]
Chitosan	Purple-fleshed sweet potato extract	Smart	+	−	N	+	−	−	N	+	N	−	−	−	[46]
Agar	Arnebia euchroma root extracts	Smart	−	−	+	−	+	+	+	+	N	−	+	N	[47]
Gelatin	Curcumin	Smart	±	±	N	+	−	+	−	−	N	−/+	+	N	[48]
k-carrageenan	Curcumin	Smart	N	N	N	+	+	−	+	−	−	−	+	+	[49]
Chitosan	Blueberry and blackberry pomace extracts	Smart	±	−	N	±	±	−	+	−	±	+	±	N	[19]
Chitosan	Alizarin	Smart	N	N	+	+	−	+	+	−	−	+	−	+	[50]

WS: water solubility, MC: moisture content, MA: moisture absorption, WCA: water contact angle, Th: thickness, TS: tensile strength, EB: elongation at break, YM: Young modulus, WVP: water vapor permeability, OP: oxygen permeability, T600: transparency, Op: opacity; NPs: nanoparticles, EOs: essential oils. N: Not analyzed. (±): variable, (+): increase, (−): decrease.

**Table 2:** Examples of the application of active packaging materials fabricated using the casting method in the food industry.

Packaging Film Matrix	Active Additives	Additive Functions	Remarks	Ref.
Chitosan	Pine needle extract (Cedrus deodara)	Antioxidant/physical/oxygen/water vapor permeability/color/microstructures	Films showed high antioxidant activity and protected oxygen-sensitive foods.	[148]
Chitosan	Flavanols (kaempferol, quercetin, myricetin)	Antimicrobial/Antioxidant/water vapor permeability/oxygen permeability/UV–vis light transmittance	Prevention of microbial growth	[149]
Poly(lactic acid)/ Poly(ε-caprolactone)	EOs (thymol, carvacrol)	Antioxidant	A PLA film impregnated with thymol and carvacrol had the best antioxidant activity.	[150]
Chitosan	Poly (vinyl alcohol)	Antimicrobial/ultraviolet blocking/morphology/mechanical properties/water solubility/hydrophilicity	Films exhibited antimicrobial activity against Escherichia coli, Staphylococcus aureus, and Candida albicans.	[32]
Polylactic acid	EOs (thymol, kesum, curry)	Antimicrobial/Morphology/functional chemistry/thermal stability/permeability	Films inhibited bacterial growth and extended shelf life of meats, fruits, and vegetable products	[151]
Sodium lactate/ whey protein isolate	ɛ-Poly lysine	Mechanical behavior/Antimicrobial	Films extended shelf-life by reduction of total flora and inhibiting lactic acid bacteria growth	[152]
Chitosan/ Carboxymethyl cellulose	ZnO nanoparticles	Antimicrobial/Physicochemical and physical properties	Films had good activity against gram-positive bacteria and fungi	[153]
Chitosan	ethyl-Nα-dodecanoyl-Larginate	Antimicrobial	Films exhibited antibacterial activity	[154]
Poly(ε-caprolactone)	Oxidized regenerated cellulose	Antimicrobial	Films reduced total colony-forming units on salami during storage.	[155]
LDPE/LLDPE	Ag/TiO_2_ nanoparticles	Antimicrobial	Nanoparticle addition improved antimildew and physicochemical properties of films.	[156]
Polyvinyl chloride	Ag nanoparticles	Antimicrobial/Antioxidant	Films inhibited bacterial growth, reduced oxidation, and extended shelf life	[157]
Sodium alginate	ZnO nanoparticles	Antimicrobial	Films reduced initial bacterial count	[158]
Whey protein isolate	Lactoferrin, Lysozyme, and the Lactoperoxidase	Antimicrobial	Films extended shelf-life by inhibiting bacterial growth	[159]

**Table 3:** Examples of studies on the utilization of smart packaging materials fabricated by the solution casting method in the food industry.

Packaging Film Matrix	Colorant Agent/Source	Trigger	Remarks	Ref.
Chitosan/ Polyvinyl alcohol (PVA)	Anthocyanin/Red cabbage	pH indicator	Additives increased tensile strength of film and provided color indication of pork spoilage during storage.	[162]
Chitosan/Starch/ Polyvinyl alcohol	Anthocyanin/Roselle calyx	pH indicator	Color changes in film provided indication of spoilage in pork.	[163]
Hydroxy propyl methylcellulose/ κ-carrageenan	Anthocyanin/Prunus maackii juice	pH indicator	Color changes in film provided indication of spoilage.	[164]
Agar/Tapioca starch	Anthocyanin/Red cabbage	pH indicator	Color changes in film provided indication of spoilage in sausage.	[165]
Cassava starch	Anthocyanin/Blueberry residue	pH indicator	Color changes in film provided indication of spoilage.	[166]
Methylcellulose/ Chitosan nanofiber	Anthocyanin/Barberry (BA)	pH indicator	Films underwent color changes when exposed to different pH conditions.	[79]
Poly vinyl pyrrolidone/CMC/Bacterial cellulose/Guar gum	Anthocyanin/Red cabbage	pH indicator	Anthocyanin addition improved physicochemical properties of films and were suitable as color sensors of pH changes.	[167]
Gelatin/Gellan gum	Anthocyanins/Red radish	pH indicator	Films underwent color changes when exposed to different pH conditions.	[168]
Chitosan/Pectin	Anthocyanin Hibiscus rosa-sinensis	pH indicator	Color changes in film provided indication of spoilage during storage.	[169]
Cellulose acetate nanofibers	Alizarin	pH indicator	Color changes in film provided indication of spoilage.	[170]
Bacterial cellulose nanofiber	Anthocyanin/Black carrot	pH indicator	Films underwent color changes when exposed to different pH conditions.	[171]
Glucomannan/Polyvinyl alcohol	Betacyanin	pH indicator	Films underwent color changes when exposed to different pH conditions.	[172]
Methylcellulose/ Chitin nanofiber	Anthocyanins/Red barberry	pH indicator	Color changes in film provided indication of spoilage in fish and meat samples during storage.	[28]
*Artemisia sphaerocephala* Krasch. gum (ASKG)/Carboxymethyl cellulose sodium	Anthocyanins/Red cabbage	pH/Gas/volatile compounds indicator (NH_3_)	Color changes in film in response to pH changes or NH_3_ production provided indication of spoilage	[132]
Polylactide/Poly hydroxybutyrate	β-carotene, Chlorophyll, Curcumin, Lutein	Temperature/Light	Color changes in film in response to changes in temperature or light exposure	[173]
Starch/Polyvinyl alcohol	Anthocyanins/Roselle	Temperature/pH indicator	Color changes in film in response to changes in pH or light exposure	[174]
Agar	Arnebia euchroma root	Temperature/Freshness	Film changed color when fish spoiled.	[47]
Chitosan/Polyvinyl alcohol	Anthocyanins/ Red cabbage	Time/Temperature	The colorimetric film on pasteurized milk shows visual color changes to consumers.	[118]
Chitosan	Chlorophyll	Temperature	Film changed color when exposed to elevated temperatures (>50 °C).	[175]
Cellulose	Anthocyanin/*Ruellia Simplex* flowers	Time/Temperature	Film changed color when exposed to different temperatures: pink/blue (at 13 °C); purplish/blue (at 25 °C); yellow/gray (at 40 °C)	[176]
Bacterial cellulose nanofibers	Anthocyanin/Black carrot	Gas/volatile ammonia compounds	Film changed color in response to gas production	[171]
Tara gum/Polyvinyl alcohol	Curcumin	Gas/volatile compounds (TVBN, NH_3_)	Film changed color in response to gas production	[177]

**Table 4:** Application of smart or active packaging materials fabricated by solution casting method to real food products tested at room or refrigerator temperature.

Food model	Polymers	Active materials	Smart or Active	Function	Remarks	Ref.
Shrimp	Bovine skin gelatin	ZnO nanoparticles/clove essential oil	Active	Antibacterial	Composite films showed antibacterial activity against *Listeria monocytogenes* and *Salmonella Typhimurium* inoculated in shrimp during refrigerated storage.	[232]
Chicken breast meat	Carboxymethyl cellulose	Okra mucilage/ ZnO nanoparticles	Active	Antimicrobial/Antioxidant	Incorporating okra mucilage and ZnO nanoparticles in films reduced microbial growth, oxidation, and gas production.	[233]
Vacuum-packed beef patties	Corn-zein-laminated linear LDPE film	Thymol, carvacrol, and eugenol essential oil	Active	Antioxidant	Incorporating essential oils in films reduced lipid oxidation and color changes in fresh ground beef patties during storage.	[234]
Pork meat	Distiller dried grains with soluble protein	Green tea, oolong tea, and black tea extracts	Active	Antioxidant	Incorporating tea extracts increased the antioxidant activity of films.	[235]
Lamb meat	Whey protein isolate/cellulose nanofibre/	TiO_2_ nanoparticle/rosemary essential oil	Active	Antimicrobial/Antioxidant	Nanocomposite films reduced total viable count, *Pseudomonas* spp, *Enterobacteriaceae*, Lactic acid bacteria, *Staphylococcus aureus*, *Listeria monocytogenes*, and *Escherichia coli* counts. Higher inhibition observed for Gram-positive than Gram-negative bacteria	[236]
Frozen blue shark (Prionace glauca)	low density polyethylene (LDPE)	Barley husk extracts	Active	Antioxidant	Hydrolytic activity and lipid oxidation are sensitive to antioxidant content and storage time.	[237]
Palm oil	Cassava starch	Mango and acerola pulp	Active	Antioxidant	Antioxidants were effective additives for protecting the packaged product.	[238]
Strawberry	Clay/PE polymer	Carvacrol and thymol essential oils	Active	Antifungal	Incorporating essential oils in films increased antifungal activity against *Botrytis*.	[128]
Tomato	Chitosan	TiO_2_ nanoparticles	Active	Gas scavenger	Nanocomposite films delayed tomato ripening.	[239]
Pear	Papaya (*Carica papaya* L.) puree	Ascorbic acid and *Moringa* leaf extract	Active	Antioxidant	Films increased shelf-life and improved sensory properties of pears.	[240]
Banana	Chitosan	*Sonneratia caseolaris* (L.) Engl. leaf extract	Active	Antimicrobial	Incorporating a leaf extract into the films increase the shelf-life of bananas	[241]
Gorgonzola cheese	Cellulose polymeric films and laminated films	Natamycin	Active	Antifungal	Incorporating the antifungal agent into film led to increased inhibition of *P. roqueforti*	[242]
Fish	Chitin nanofiber/methylcellulose	Red barberry anthocyanins (RBAs)	Active/Smart	Antimicrobial/Antioxidant/ Colorimetric	Films exhibited good antioxidant and antimicrobial activity, as well as ability to detect quality changes.	[28]
Chicken	Chitosan/corn starch	Hibiscus rosa-sinensis anthocyanin	Smart	Colorimetric	Films exhibited good optical and morphological properties and are sensitive to pH changes.	[169]
Sausage	Agar/Tapioca starch	Red cabbage anthocyanin	Smart	Colorimetric	Anthocyanins change color in response to quality changes in sausage during storage.	[165]
Chicken	Cassava starch	Blueberry residue anthocyanin	Smart	Colorimetric	Anthocyanins change color in response to pH (quality) changes in chicken during storage.	[166]
Pork/Fish	Chitosan	Bauhinia blakeana Dunn. flower anthocyanin	Smart	Colorimetric	Anthocyanins change color in response to quality changes in pork and fish during storage.	[204]
Lamb meat	Chitosan nanofibers/methylcellulose	Saffron petal anthocyanins	Active/Smart	Antimicrobial/Antioxidant/Colorimetric	Chitosan provides antimicrobial activity while anthocyanins provide antioxidant activity and change color in response to changes in lamb quality during storage.	[42]
Red meat	Methylcellulose/chitosan nanofiber	Barberry anthocyanin	Active/Smart	Antioxidant/Colorimetric	Chitosan provides antimicrobial activity while anthocyanins change color in response to changes in meat quality during storage.	[79]
Banana	PVA/glucomannan	Sappan Wood extracts	Smart	Antioxidant	The wood extract changed color in response to quality changes in banana during storage.	[243]
Milk	Starch/Polyvinyl alcohol	Purple sweet potato anthocyanin	Smart	Antimicrobial/Colorimetric	The anthocyanins gave a color change in response to alterations in milk quality. The films also exhibited antimicrobial activity against *Aspergillus niger*, *Bacillus subtilis*, and *Staphylococcus aureus*.	[244]

## Data Availability

Not applicable.
